# [Corrigendum] Upregulation of interleukin‑17F in colorectal cancer promotes tumor invasion by inducing epi​thelial‑mesenchymal transition

**DOI:** 10.3892/or.2025.8885

**Published:** 2025-03-12

**Authors:** Yusheng Chen, Zhou Yang, Dejun Wu, Zhijun Min, Yingjun Quan

Oncol Rep 42: 1141–1148, 2019; DOI: 10.3892/or.2019.7220

Subsequently to the publication of the above paper, an interested reader drew to the authors' and the Editor's attention that the clone image featured in Fig. 4A for the ‘NC’ (or ‘negative control’) data panel on p. 1146 had also been featured as the ‘NC’ experimental data panel in Fig. 4A of another article entitled ‘Interleukin 1β/1RA axis in colorectal cancer regulates tumor invasion, proliferation and apoptosis via autophagy’ (DOI: 10.3892/or.2020.7475), which was published by the same research group in the same journal.

In this pair of articles, the authors were researching IL-17F and IL-1B/1RA, respectively; all experiments were performed on interleukins using the same batch of cells, and it was the authors’ intention to have included these data, as they were portrayed, in both articles. However, to avoid any possible confusion, the authors now present alternative data for the ‘NC’ experiment in Fig. 4A for the above article, and the revised figure is shown on the next page.

In addition, the authors have realized that the data panel in Fig. 1C on p. 1144, which was intended to represent the ‘Tumor’ immunohistochemical experiment (right-hand panel), was inadvertently chosen incorrectly. The correct data for this panel is shown in the revised version of Fig. 1 opposite. Note that this error did not affect either the results or the conclusions reported in this paper. The authors are grateful to the Editor of *Oncology Reports* for allowing them the opportunity to publish this Corrigendum, and they apologize to the Editor and to the readership for any inconvenience caused.

## Figures and Tables

**Figure 1.**
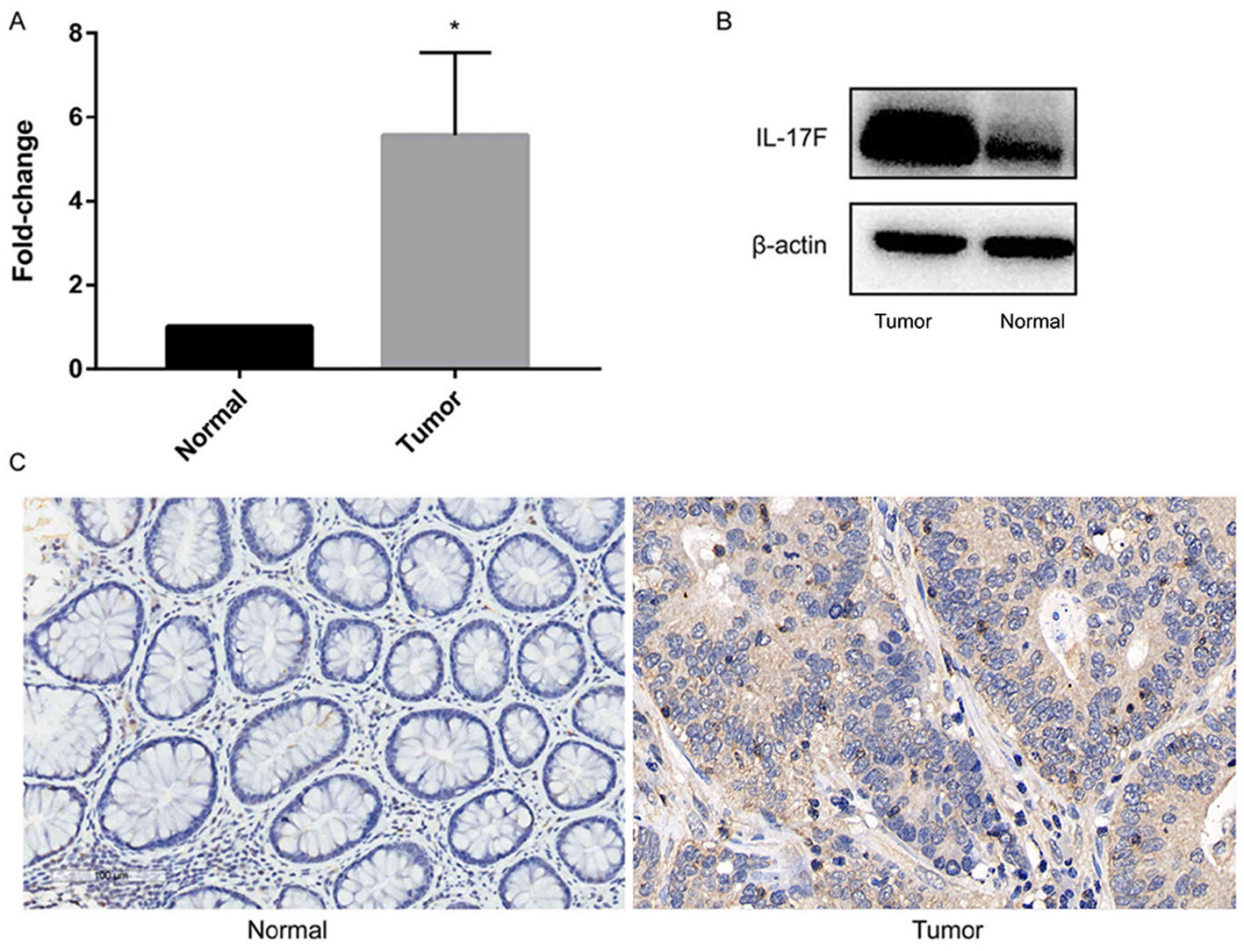
IL-17F is overexpressed in colorectal cancer. (A) Expression of common IL-17F was detected by reverse transcription-quantitative PCR. *P<0.05, compared to the normal tissues. (B) Expression of IL-17F protein was detected by western blotting. (C) Expression of IL-17F protein was detected by immunohistochemistry. IL-17F, interleukin-17F.

**Figure 4.**
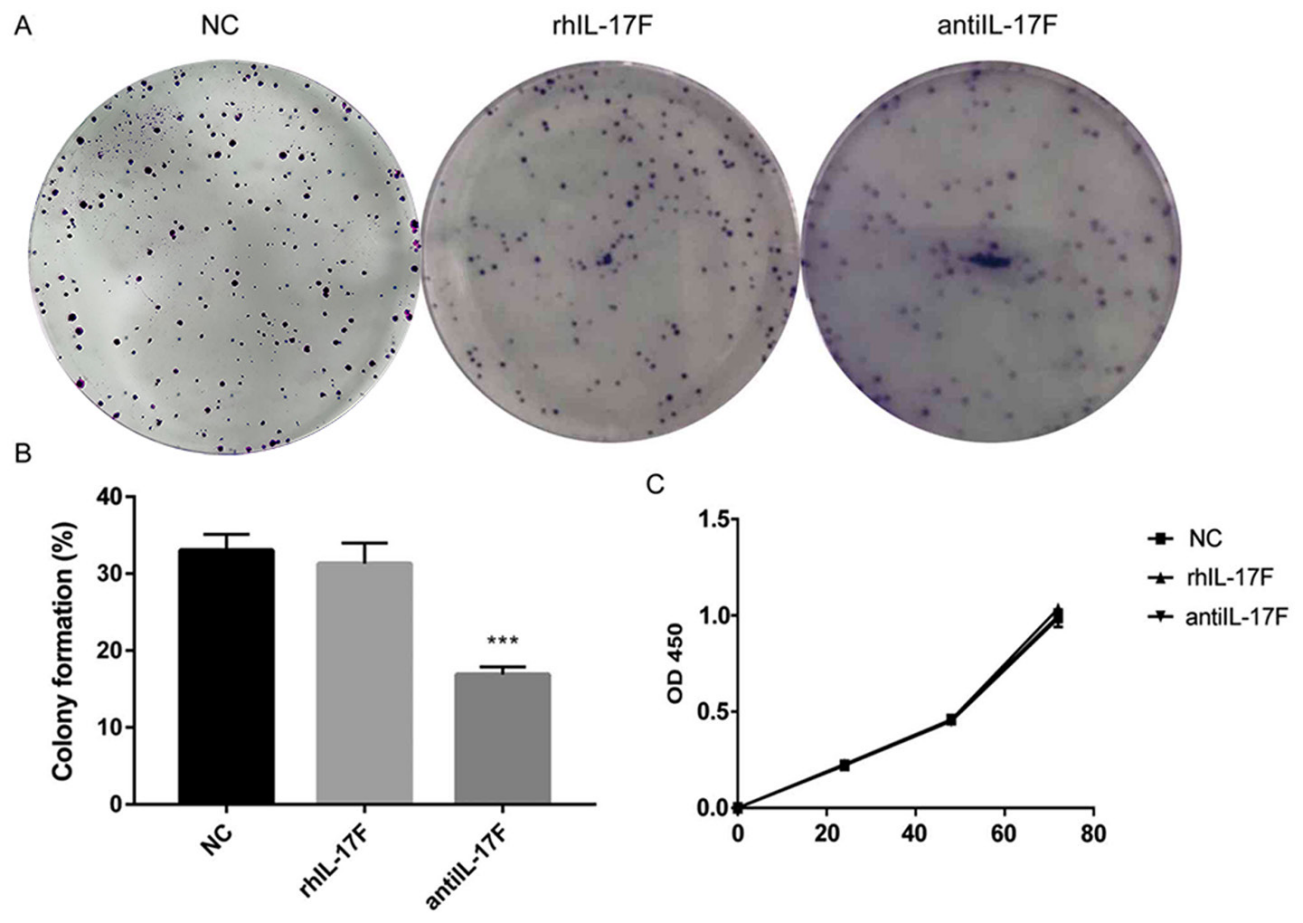
(A) Colony formation assay for each group. (B) The group treated with anti-interleukin-17F antibody exhibited decreased percentage of colony formation compared with the negative control (NC) group. ***P<0.001. (C) Cell proliferation was detected by Cell Counting Kit-8 assay, and no difference was observed. rhIL-17F, recombinant human interleukin-17F.

